# Comprehensive Pan-Cancer Analysis Confirmed That ATG5 Promoted the Maintenance of Tumor Metabolism and the Occurrence of Tumor Immune Escape

**DOI:** 10.3389/fonc.2021.652211

**Published:** 2021-03-25

**Authors:** Chunxiao Xu, Yusheng Zang, Yuxiang Zhao, Weiqiang Cui, Hong Zhang, Yingcui Zhu, Man Xu

**Affiliations:** ^1^ Department of Cardiology, Sunshine Union Hospital, Weifang, China; ^2^ School of Medicine, Kunming University of Science and Technology, Kunming, China; ^3^ Institute of Bioengineering, Biotrans Technology Co., LTD, Shanghai, China; ^4^ United New Drug Research and Development Center, Biotrans Technology Co., LTD, Ningbo, China; ^5^ Department of Anesthesiology, Sunshine Union Hospital, Weifang, China; ^6^ Department of Internal Medicine, Weifang Maternal and Child Health Hospital, Weifang, China; ^7^ Department of Radiology, Jinan Central Hospital, Jinan, China; ^8^ School of Medicine, Shandong University, Jinan, China

**Keywords:** pan-cancer, autophagy-related protein 5 (Atg5), autophagy, tumor metabolism, tumor immune escape

## Abstract

**Background:**

Autophagy related protein 5 (ATG5) is an important autophagosome formation related protein, and its involvement in the biological process of autophagy has been shown to correlate with tumor metabolic patterns and the formation of tumor heterogeneity. However, the role of ATG5 in tumor metabolism and tumor immunity remains unclear.

**Method:**

In order to explore this problem, this study was designed to reveal the role of ATG5 in tumor metabolism and tumor immunity through pan-cancer analysis of multi-database. GTEx database, CCLE database, and TCGA database were used to describe the expression, prognosis, immune microenvironment, immune new antigen, immune checkpoint, TMB, and microsatellite instability of ATG5 in 33 types of tumors. A series of bioinformatics tools and methods were used for quantitative analysis and panoramic description, such as to Estimate, Scanneo and GSEA.

**Result:**

The differential analysis results of multiple databases showed that ATG5 was ubiquitously highly expressed in pan-cancer, especially in solid tumors. Survival analysis revealed that ATG5 was universally associated with the prognosis of pan-cancer, and high ATG5 expression was significantly associated with poor patient prognosis in most cases. Further, the expression level of ATG5 was confirmed to be associated with tumor immune infiltration and tumor microenvironment, especially in BRCA, KIRC, and LIHC. In addition to this, ATG5 expression was confirmed to correlate with these clinically significant phenotypes, in conjunction with immune neoantigens and immune checkpoint gene expression profiles in pan-cancer. In addition to TMB and microsatellite instability in pan-cancer, we confirmed that ATG5 expression affects the expression of DNA repair genes and methyltransferases in pan-cancer, and found through gene set enrichment analysis that ATG5 is involved in the regulation of numerous signaling pathways involved in cancer metabolism and cancer immunity.

**Conclusions:**

ATG5 participated in the formation of autophagosomal membrane important molecule LC3-II outside, and played an important role in tumor metabolism and tumor immunity. The comprehensive pan-cancer analysis not only revealed the potential of ATG5 in tumor-targeted therapy but also suggested ATG5 as a promising tumor predictive biomarker in most solid tumors.

## Introduction

Autophagy related protein 5 (ATG5) is an important autophagosome formation related protein that functions as an E1-like activating enzyme in eukaryotic cells (1, 2). In addition to LC3-II, an important molecule involved in autophagosomal membrane formation through the ATG5-ATG12/ATG16 complex, ATG5 has been shown to play important roles in viral infection (3, 4), tumor apoptosis (5, 6), and tumor proliferation (7, 8). And several reports have suggested that ATG5 is promising as a novel target for clinical cancer therapy.

In recent years, autophagy has been shown to correlate with tumor metabolic patterns and the formation of tumor heterogeneity (9). On the one hand, as an important pathway of cellular material recycling and energy metabolism, autophagy helps tumor cells to escape from the attack of high-levels of ROS generated by aerobic glycolysis by degrading damaged mitochondria, which guarantees the sustainability of the Warburg effect and the metabolic pattern of tumors. On the other hand, antagonism of autophagy with inflammation decreased the degree of chronic inflammatory infiltration and repressed the inflammatory cancer transformation process. These biological processes, which influence cell fate decisions, conspire at distinct effects of tumor stromal cells and versus tumor immune cells to program tumor metabolic patterns and shape the tumor immune landscape (10, 11).

Unfortunately, the role of ATG5, as an important autophagy-related molecule, in tumor metabolism versus tumor immunity remains obscure. Therefore, this study plans to reveal it by pan-cancer analysis of the combined multi-database. In recent years, the rise of high-throughput sequencing based cancer atlas initiatives with omics technologies has provided a new perspective in cancer research. The use of transcriptome technology to reveal the role of gene expression in tumor cells has long been appreciated, and emerging deconvolution network algorithms allow tumor investigators to extract the expression profiles of immune cells from transcriptome data and describe their distribution patterns. In addition to this, the vast amount of data generated by the unprecedented bioinformatics revolution can be used to delineate the panoramic landscape covering all known genes and cancer types (12). This bioinformatic analysis that uses multiple databases to analyze the expression, prognosis, mutational pattern, and function of a gene in different tumors is called pan-cancer analysis, and it can be used to investigate the roles and connections of genes in different tumors (13).

In this study, we utilized a pan-cancer analysis to analyze the association of ATG5 expression, prognosis, immune microenvironment, immune neoantigens, immune checkpoints genes, TMB, and microsatellite instability in 33 tumors. Confirmed that ATG5 expression affects the expression of DNA repair genes and methyltransferases in Pan-cancer, and found that ATG5 is involved in the regulation of signaling pathways involved in cancer metabolism and tumor immunity by gene set enrichment analysis.

## Materials and Methods

### Sample Information

The gene expression matrix and clinical information data in each tumor and normal were obtained from the GTEx database (https://gtexportal.org/) and TCGA database (https://portal.gdc.cancer.gov/). Among them, 31 cancers were included in gene expression analysis with GTEx and 27 cancers were included in TCGA integrated analysis with GTEx. The expression data of each tumor cell line were downloaded from the CCLE database (https://portals.broadinstitute.org/). The pan-cancer immune infiltrating cell score data were downloaded from the timer database (https://cistrome.shinyapps.io/timer/). Tumor name abbreviations and corresponding meanings are given below: ACC(Adrenocortical carcinoma); BLCA(Bladder Urothelial Carcinoma); BRCA(Breast invasive carcinoma); CESC(Cervical squamous cell carcinoma and endocervical adenocarcinoma); CHOL(Cholangiocarcinoma); COAD(Colon adenocarcinoma); COAD (Colon adenocarcinoma); READ (Rectum adenocarcinoma Esophageal carcinoma); DLBC(Lymphoid Neoplasm Diffuse Large B-cell Lymphoma); ESCA(Esophageal carcinoma); GBM(Glioblastoma multiforme); HNSC(Head and Neck squamous cell carcinoma); KICH(Kidney Chromophobe); KIRC(Kidney renal clear cell carcinoma); KIRP(Kidney renal papillary cell carcinoma); LAML(Acute Myeloid Leukemia); LGG(Brain Lower Grade Glioma); LIHC(Liver hepatocellular carcinoma); LUAD(Lung adenocarcinoma); LUSC(Lung squamous cell carcinoma); MESO(Mesothelioma); OV(Ovarian serous cystadenocarcinoma); PAAD(Pancreatic adenocarcinoma); PCPG(Pheochromocytoma and Paraganglioma); PRAD(Prostate adenocarcinoma); READ(Rectum adenocarcinoma); SARC(Sarcoma); SKCM(Skin Cutaneous Melanoma); STAD(Stomach adenocarcinoma); STES(Stomach and Esophageal carcinoma); TGCT(Testicular Germ Cell Tumors); THCA(Thyroid carcinoma); THYM(Thymoma); UCEC(Uterine Corpus Endometrial Carcinoma); UVM(Uveal Melanoma).

### Expression Analysis of Autophagy-Related Protein 5 in Pan-Cancer

Differences in ATG5 expression levels in tumor tissues and normal tissues were analyzed by edgeR software. The Kruskal Wallis test was used to analyze the expression levels of ATG5 in different normal tissues and different tumor cell lines. Violin plots were drawn by the R package ggplot.

### Prognostic Analysis of Autophagy-Related Protein 5 in Pan-Cancer

Univariate survival analysis was used to analyze the correlation of ATG5 expression with patient survival. The Kaplan-Meier method was used to compare survival with different levels of ATG5 expression. The expression levels of ATG5 in tumors and adjacent noncancerous tissues were divided into ATG5 high expression and ATG5 low expression groups by a bipartite method. Univariate Cox survival analysis was done by survival software. Visualization was done by software forestplot.

### Association Analysis of Autophagy-Related Protein 5 With the Immune Microenvironment

Tumor infiltrating lymphocytes are independent predictors of sentinel lymph node status and survival in cancer, with the immune cell score of the respective tumor sample described by the immune score and stromal score. Correlation of gene expression with immune cell scores was analyzed using the software estimate and considered significant and positive when p<0.05 and R>0.20.

### Association Analysis of Autophagy-Related Protein 5 With Immune Neoantigens and Immune Checkpoints Genes

Neoantigen encoded by a mutated gene in tumor cells, coming from biological events such as point mutations, deletion mutations, and gene fusions. Scanneo calculates its binding affinity score using antigenic epitopes with a length of 8~11 amino acids, while epitopes with a score less than 500 nm are reported as neoantigens. Predicted neoantigens were then ranked according to binding affinity, variant allele frequency, and antigenicity index values. The number of neoantigens per tumor sample was counted separately using scanneo, and the relationship between ATG5 expression and the number of antigens was analyzed. Further, the common more than 40 immune checkpoint genes were analyzed for their expression relationship with ATG5, these immune checkpoint genes were extracted separately, and the correlation with ATG5 expression was calculated. Correlations were considered significant and positive when p<0.05 and R>0.20.

### Association Analysis of Autophagy-Related Protein 5 With Tumor Mutational Burden and Microsatellite Instability

Tumor mutational burden (TMB), as a quantifiable biomarker, can be used to reflect the number of mutations contained in a tumor cell. The TMB of each tumor sample was counted separately using Spearman’s rank correlation coefficient. Microsatellite instability (MSI) refers to the occurrence of a new microsatellite allele phenomenon when compared with normal tissue, in a tumor, any alteration in the length of a microsatellite caused by an insertion or deletion of a repeat unit. Correlation of ATG5 expression with MSI was analyzed using Spearman’s rank correlation coefficient.

### Association Analysis of Autophagy-Related Protein 5 With DNA Mismatch Repair Genes and Methyltransferases

Mismatch repair is an intracellular mismatch repair mechanism, and loss of function of key genes of this mechanism results in DNA replication errors that cannot be repaired, which in turn leads to the generation of higher levels of somatic mutations. The correlation of five MMRs genes (MLH1, MSH2, MSH6, PMS2, EPCAM) with ATG5 expression was assessed using the expression profile data from TCGA. DNA methylation is a form of chemical modification of DNA capable of altering epigenetic inheritance and controlling gene expression without altering the DNA sequence. Here we analyzed the correlation between ATG5 expression and the expression of four methyltransferases. Visualization analysis was done by ggplot. Correlations were considered significant and positive when p<0.05 and R>0.20.

### Gene Set Enrichment Analysis of Autophagy-Related Protein 5 in Pan-Cancer

Gene set enrichment analysis (GSEA) is an analytical method that compares genes with predefined gene sets to explore their expression status within a specific functional gene set and whether such expression status is associated with a biological process, molecular function, or cellular component in some way statistically significant (14). Kyoto Encyclopedia of Genes and Genomes (KEGG) is a comprehensive database incorporating genomic, chemical, and systematic functional information. Another dataset used for GSEA analysis is the molecular signatures database (MsigDB) (15), where the Hallmark gene set was used in this analysis. Using |NES| > 1, p-value < 0.05, FDR < 0.25 as the threshold of GSEA, pathways were considered significantly enriched when they met the sub-conditions.

## Results

### Autophagy-Related Protein 5 Is Highly Expressed in Pan-Cancer

**Figure 1:**
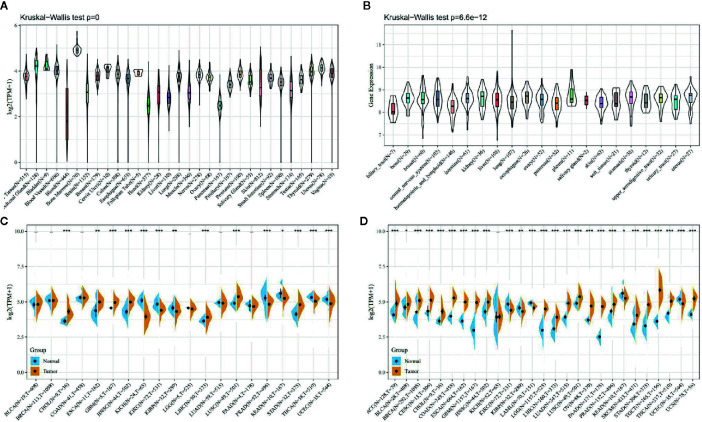
ATG5 expression in pan-cancer. **(A)** Expression levels of ATG5 in a dataset containing 31 tissues, derived from the GTEx. **(B)** Expression levels of ATG5 in a dataset containing 21 tissue in tumor cell lines, derived from the CCLE. **(C)** Expression levels of ATG5 in tumor and paired adjacent noncancerous tissues containing 20 tissues from TCGA, *P < 0.05, **P < 0.01, ***P < 0.001. **(D)** ATG5 expression difference in 27 tumors integrating data of normal tissues in GTEx database and data of TCGA tumor tissues, *P < 0.05, **P < 0.01, ***P < 0.001.

As shown in **Figure 1A**, first we analyzed the expression levels of ATG5 in various tissues by utilizing the GTEx datasets, describing the expression pattern of ATG5 in 31 tissues. As shown in **Figure 1B**, the expression levels of ATG5 in 21 tissue cell lines were analyzed according to tissue origin using the data of individual tumor cell lines downloaded from the CCLE database. Next, as shown in **Figure 1C**, we retrieved the differential expression pattern of ATG5 in cancer vs. adjacent carcinoma in individual tumor samples from the TCGA database. As shown in **Figure 1D**, considering that there were fewer normal samples in TCGA, we integrated the data of normal tissues in the GTEx database and the data of TCGA tumor tissues to perform the expression difference analysis of ATG5 in 27 tumors. From the expression analysis of ATG5 in Pan-cancer, it can be found that the expression level of ATG5 is generally higher in almost all solid tumors than that in normal tissues.

### Autophagy-Related Protein 5 Is Associated With Prognosis in Pan-Cancer

**Figure 2:**
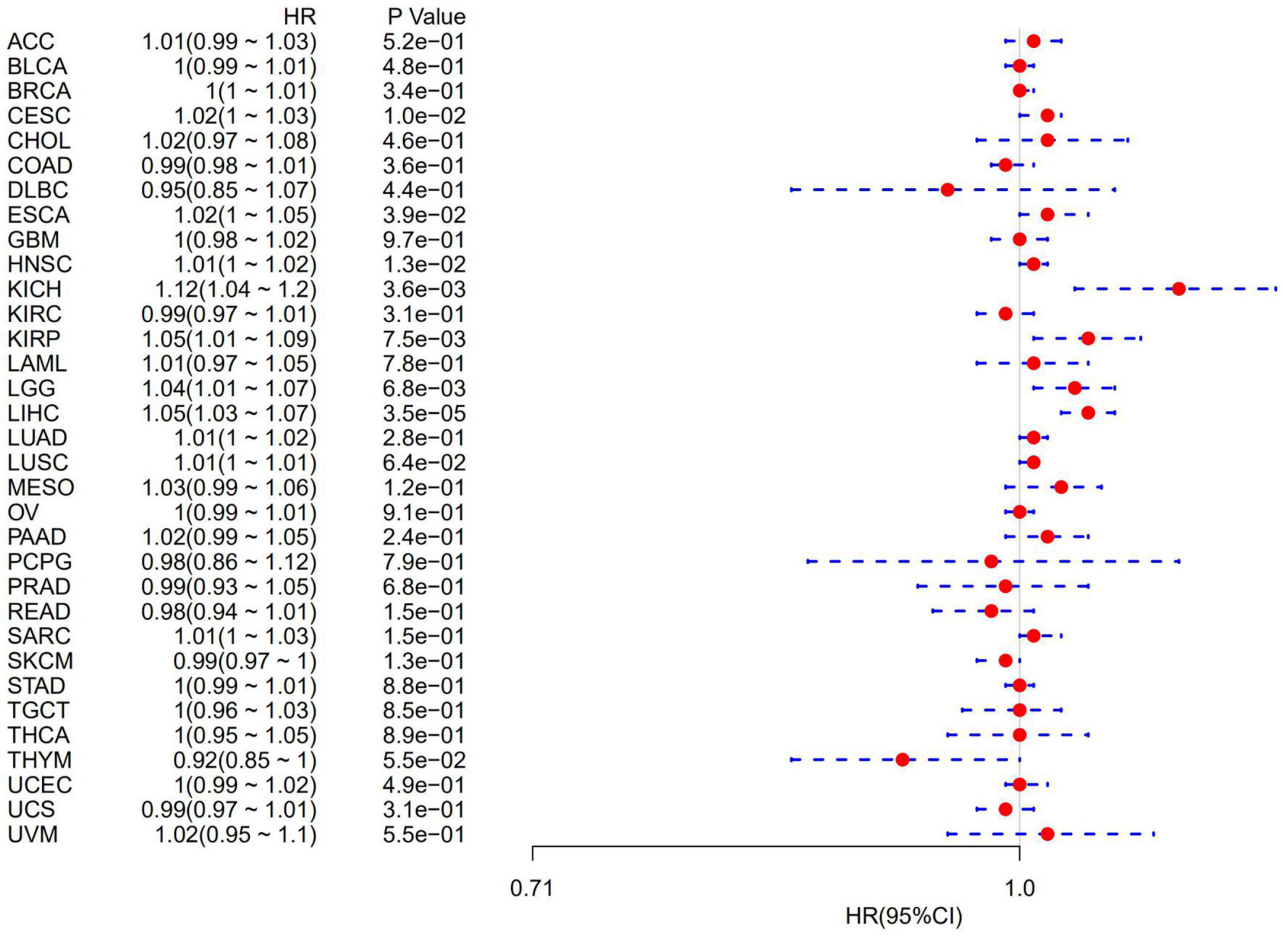
Forest plot of the relationship between ATG5 expression and overall survival time in days, using univariate survival analysis, across 33 tumors.

**Figure 3:**
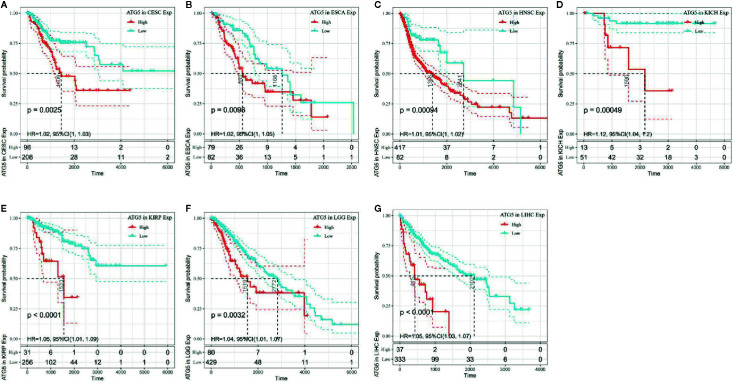
Kaplan–Meier OS curves of ATG5 expression in the seven most significantly associated tumors. **(A)** KM plot of high and low ATG5 expression in CESC patients. **(B)** KM curves of high and low ATG5 expression in ESCA patients. **(C)** KM plot of high and low ATG5 expression in HNSC patients. **(D)** KM curves of high and low ATG5 expression in KICH patients. **(E)** KM curves of high and low ATG5 expression in KIRP patients. **(F)** KM curves of high and low ATG5 expression in LGG patients. **(G)** KM curves of high and low ATG5 expression in LIHC patients.

The association of ATG5 expression with overall survival was first calculated for 33 tumors in TCGA using univariate survival analysis. As shown in **Figure 2**, the forest plots among the 33 tumors were showed that ATG5 could significantly affect the overall survival of CESC (HR = 1.02, P = 0.01), ESCA (HR = 1.02, P = 0.039), HNSC (HR = 1.12, P = 0.013), KICH (HR = 1.12, P = 0.0036), KIRP (HR = 1.05, P = 0.0075), LGG (HR = 1.04, P = 0.0068), and LIHC (HR = 1.05, P < 0.001) patients. These tumors all suggested that ATG5 was associated with poor patient outcomes, especially in KICH. The KM curves for tumors in which ATG5 expression was significantly associated with the patient outcome are shown in **Figure 3**. The results showed that high expression of ATG5 was significantly associated with poor prognosis of patients, which suggested that ATG5 may be a potential prognostic indicator molecule in pan-cancer.

### Autophagy-Related Protein 5 Is Correlated With Tumor Immune Infiltration and Tumor Microenvironment in Pan-Cancer

**Figure 4:**
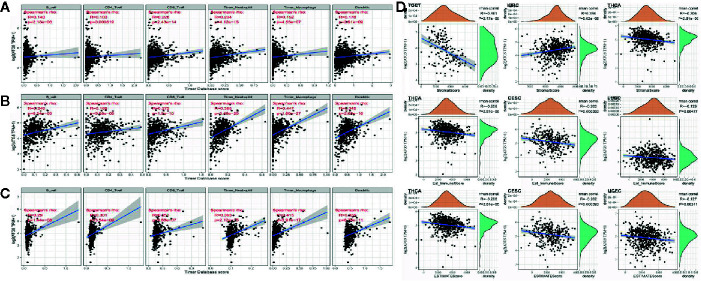
Correlation analysis between ATG5 expression in Pan-cancer and tumor immune infiltration and tumor microenvironment. **(A)** Correlation analysis between expression levels of ATG5 and immune cell infiltration in BRCA. **(B)** Correlation analysis between expression levels of ATG5 and immune cell infiltration in KIRC. **(C)** Correlation analysis between the expression level of ATG5 and immune cell infiltration in LIHC. **(D)** Correlation analysis between ATG5 expression in Pan-cancer and immune score, between ATG5 expression and stromal score, and between ATG5 expression and estimate immune score.

Tumor infiltrating lymphocytes are independent predictors of sentinel lymph node status and survival in cancer, and immune infiltration analysis confirmed that ATG5 expression correlates with the level of immune infiltration in different types of cancer, especially in BRCA, KIRC, and LIHC. As shown in **Figures 4A, B**, ATG5 expression levels were significantly correlated with those of CD8^+^ T cells (R= 0.228, P < 0.001) and neutrophils (R = 0.228, P < 0.001) in BRCA, and B cells (R = 0.249, P < 0.001), CD8^+^ T cells (R = 0.272, P < 0.001), neutrophils (R = 0.385, P < 0.001) and dendritic cells (R = 0.345, P < 0.001) in KIRC. As shown in **Figure 4C**, ATG5 expression was significantly correlated with all six tumor infiltrating lymphocytes in LIHC, B cells (R = 0.29, P < 0.001), CD4^+^ T cells (R = 0.301, P < 0.001), CD8^+^ T cells (R = 0.251, P < 0.001), neutrophils (R = 0.393, P < 0.001), macrophages (R = 0.415, P < 0.001), and dendritic cells (R = 0.331, P < 0.001). In addition, to explore the roles that the ATG5 influenced tumor immune microenvironment has during tumor development, we analyzed the immune score and stromal score of individual tumor samples using the R package Estimate, as shown in **Figure 4D**, the top three tumors with the most significant correlation between ATG5 expression and immune score among the 33 tumors were TGCT (R = -0.461, P < 0.001), KIRC (R = 0.234, P < 0.001), and THCA (R = -0.206,P < 0.001), the top three tumors whose ATG5 expression was most significantly correlated with stromal score were THCA (R = -0.206, P < 0.001), CESC (R = -0.202, P < 0.001) and LUSC (R = -0.126, P < 0.005), and the relationships between ATG5 expression and the immune score of estimate were THCA (R = -0.206, P < 0.001), CESC (R = -0.202, P < 0.001) and LUSC (R = -0.127, P < 0.005). These results suggested that in the tumor immune microenvironment, the expression levels of ATG5 in TGCT and THCA are significantly negatively correlated with the immune score, but positively correlated with KIRC, THCA, CESC, and LUSC. And the expression levels of ATG5 in THCA and CESC are significantly negatively correlated with the estimate immune score.

### Autophagy-Related Protein 5 Expression in Pan-Cancer Is Associated With Immune Neoantigens and Immune Checkpoint Genes

**Figure 5:**
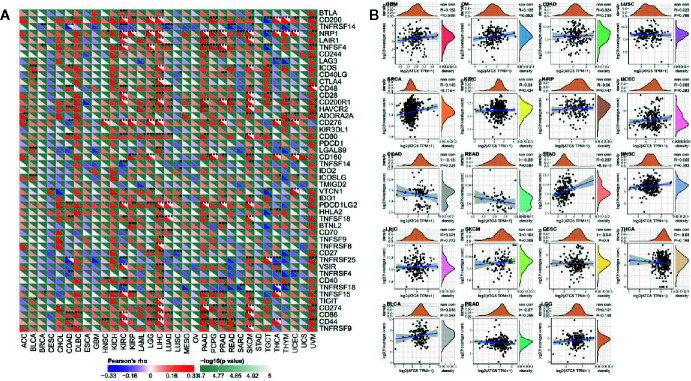
Correlation analysis between ATG5 expression and immune neoantigens and immune checkpoint genes in pan-cancer. **(A)** Correlation analysis between ATG5 expression in Pan-cancer and immune checkpoint gene expression. *Significant correlation P < 0.05, **Significant correlation P < 0.01, ***Significant correlation P < 0.001. **(B)** Correlation analysis between ATG5 expression in pan-cancer and the number of tumor neoantigens in 19 types of tumors.

As shown in **Figure 5A**, the relationship of ATG5 expression and the expression of immune checkpoint genes could be probed by expression data of more than forty immune checkpoint genes commonly found in various types of tumors. Our results showed that the expression of ATG5 was positively correlated with the expression levels of immune checkpoint genes in various types of tumors, such as KIRC, LIHC, and UVM, suggesting that in some tumors ATG5 may play a role in modulating the pattern of tumor immunity by regulating the expression levels of these immune checkpoint genes. As shown in **Figure 5B**, we counted the number of neoantigens for each tumor type separately and analyzed the relationship between ATG5 expression and the number of these neoantigens, and found that only in STAD was the expression of ATG5 positively correlated with the number of neoantigens (R = 0.287, P < 0.01).

### Autophagy-Related Protein 5 Expression Is Associated With Tumor Mutational Burden and Microsatellite Instability in Pan-Cancer

**Figure 6:**
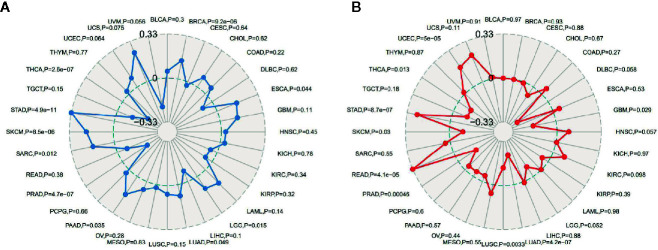
Correlation analysis between ATG5 expression in pan-cancer and TMB, MSI. **(A)** Results of correlation analysis between ATG5 expression in pan-cancer and TMB described using Spearman’s rank correlation coefficient. **(B)** Results of correlation analysis between ATG5 expression in pan-cancer and MSI described using Spearman’s rank correlation coefficient.

TMB, usually measured as the number of somatic mutations occurring at an average of 1M bases in the coding regions of the tumor cell genome, is sometimes directly represented by the total number of nonsynonymous mutations, and the mutation types mainly include various forms of mutations such as single nucleotide variations (SNVs) and small insertions/deletions (Indels). TMB was used to reflect the number of mutations contained in tumor cells, is a quantifiable biomarker. As shown in **Figure 6A**, the correlation of TMB with ATG5 expression was statistically analyzed for each tumor type separately, using Spearman’s rank correlation coefficient. Notably, ATG5 expression was positively correlated with TBM in BRCA, ESCA, PAAD, SARC, SKCM, and STAD, and negatively correlated with THCA and PRAD. MSI refers to the occurrence of a new microsatellite allele in a tumor compared with normal tissue due to any alteration in the length of a microsatellite caused by insertion or deletion of a repeat unit. As shown in **Figure 6B**, the expression of ATG5 was analyzed for correlation with MSI using Spearman’s rank correlation coefficient. The results showed that ATG5 expression was significantly positively correlated with MSI in READ, STAD and UCEC, and negatively correlated with LUAD and PRAD.

### Autophagy-Related Protein 5 Affects DNA Mismatch Repair Genes and Methyltransferase Expression in Pan-Cancer

**Figure 7:**
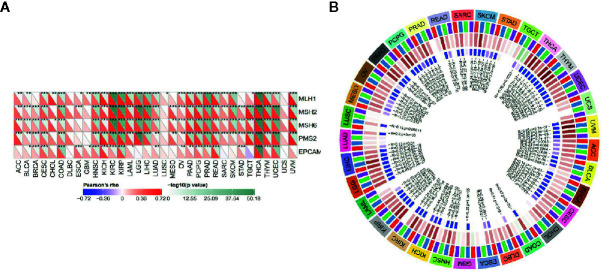
Correlation analysis between the expression of ATG5 in pan-cancer and the expression levels of DNA repair genes and methyltransferases. **(A)** Correlation analysis between gene expression levels of the five MMRs and ATG5 expression. **(B)** Correlation analysis between ATG5 expression and the expression of four methyltransferases. DNMT1 is colored red, DNMT2 was colored blue, DNMT3a was colored green, and DNMT3b is colored purple.

As shown in **Figure 7A**, almost all the MMRs genes were positively correlated with the expression level of ATG5 except CHOL, DLBC, LUSC and USC, which suggested that ATG5 could maintain tumor cell viability by upregulating DNA mismatch repair related genes. DNA methylation is the action of DNA methyltransferases that covalently bond a methyl group at the 5’ carbon position of cytosine of genomic CpG dinucleotides. As shown in **Figure 7B**, through the correlation visualization analysis between ATG5 expression and the expression of the four methyltransferases, we obtained a significant positive correlation between ATG5 expression levels and methyltransferase expression levels in all tumors, which suggested that ATG5 could mediate tumorigenesis and progression by regulating the epigenetic status in human pan-cancer.

### Autophagy-Related Protein 5 Is Implicated in the Regulation of Signaling Pathways Involved in Cancer Metabolism and Tumor Immunity

**Figure 8:**
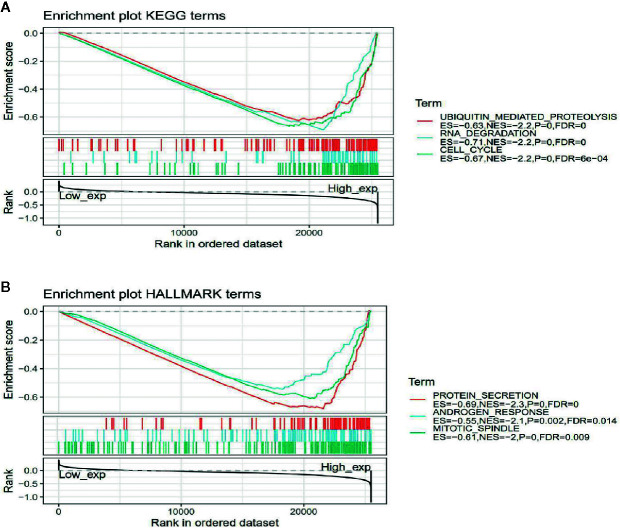
Gene set enrichment analysis of ATG5 associated with signaling pathways in KEGG and hallmark datasets. **(A)** Results of GSEA of ATG5 ranked in the top 3 for its correlation with signaling pathways in KEGG database. **(B)** Results of GSEA of the top 3 rankings of ATG5 correlation with signaling pathways in hallmark dataset.

To observe the effect of gene expression on tumors, we divided the human pan-cancer samples into two groups with high and low expression according to the expression levels of ATG5 and analyzed the enrichment of signaling pathways in KEGG and hallmark in high and low expression groups by GSEA, as shown in **Tables 1** and **2**. The top 20 most enriched signaling pathways or biological processes according to the NES score permutation have been previously characterized Listed. As shown in **Figure 8**, the top 3 signaling pathways most significantly enriched in both databases have been listed, and notably, ubiquitin-mediated proteolysis and protein secretion were described as the most enriched signaling pathways, respectively. In addition, the KEGG pathways in p53 signaling, pancreatic cancer, mismatch repair, nucleotide excision repair, pyrimidine metabolism, small cell lung cancer, renal cell carcinoma, chronic myeloid leukemia, and pathways in cancer were described as the most enriched KEGG pathways, glycolysis, PI3K-Akt-mTOR signaling, mTORC1 signaling, DNA repair, Myc targets, adipogenesis, TGF-beta signaling pathway, IL2-STAT5 signaling pathway And oxidative phosphorylation and other biological processes were described as hallmark signaling pathways with the greatest enrichment, these results indicate that ATG5 is widely involved in the regulation of signaling pathways involved in tumor metabolism and tumor immunity.

**Table 1:** The information of KEGG terms from top 20 GSEA enrichment analysis.

Term	ES	NES	NP	FDR	FWER
KEGG_UBIQUITIN_MEDIATED_PROTEOLYSIS	−0.6304	−2.2346	0	0	0
KEGG_RNA_DEGRADATION	−0.7093	−2.1876	0	0	0
KEGG_CELL_CYCLE	−0.6696	−2.1501	0	6.00E−04	0.001
KEGG_OOCYTE_MEIOSIS	−0.5741	−2.1076	0	0.0046	0.01
KEGG_BASAL_TRANSCRIPTION_FACTORS	−0.6895	−2.0924	0	0.0055	0.014
KEGG_P53_SIGNALING_PATHWAY	−0.5559	−2.066	0	0.0068	0.022
KEGG_PANCREATIC_CANCER	−0.5716	−2.0495	0	0.0073	0.027
KEGG_SNARE_INTERACTIONS_IN_VESICULAR_TRANSPORT	−0.6174	−2.0429	0	0.0065	0.028
KEGG_MISMATCH_REPAIR	−0.7943	−2.0042	0	0.0115	0.042
KEGG_NUCLEOTIDE_EXCISION_REPAIR	−0.676	−2.0016	0	0.0105	0.042
KEGG_PYRIMIDINE_METABOLISM	−0.5598	−1.9809	0	0.0145	0.062
KEGG_PROGESTERONE_MEDIATED_OOCYTE_MATURATION	−0.5532	−1.9652	0	0.0166	0.073
KEGG_SMALL_CELL_LUNG_CANCER	−0.5345	−1.9467	0.002	0.02	0.089
KEGG_ENDOCYTOSIS	−0.5005	−1.9456	0.0039	0.0187	0.089
KEGG_PATHOGENIC_ESCHERICHIA_COLI_INFECTION	−0.5852	−1.9444	0	0.0176	0.09
KEGG_PURINE_METABOLISM	−0.4763	−1.921	0.0021	0.0204	0.107
KEGG_RENAL_CELL_CARCINOMA	−0.5434	−1.9155	0.004	0.02	0.112
KEGG_CHRONIC_MYELOID_LEUKEMIA	−0.547	−1.9153	0	0.019	0.113
KEGG_NEUROTROPHIN_SIGNALING_PATHWAY	−0.5075	−1.9152	0	0.018	0.113
KEGG_PATHWAYS_IN_CANCER	−0.4556	−1.9147	0	0.0175	0.115

**Table 2:** The information of HALLMARK terms from top 20 GSEA enrichment analysis.

Term	ES	NES	NP	FDR	FWER
HALLMARK_PROTEIN_SECRETION	−0.6895	−2.2959	0	0	0
HALLMARK_ANDROGEN_RESPONSE	−0.5531	−2.0535	0.002	0.0136	0.015
HALLMARK_MITOTIC_SPINDLE	−0.6138	−2.048	0	0.009	0.015
HALLMARK_GLYCOLYSIS	−0.5373	−2.0384	0.0019	0.0078	0.016
HALLMARK_PI3K_AKT_MTOR_SIGNALING	−0.5492	−2.0167	0	0.0088	0.021
HALLMARK_G2M_CHECKPOINT	−0.7009	−1.9931	0.0019	0.0093	0.029
HALLMARK_E2F_TARGETS	−0.7352	−1.992	0.0018	0.008	0.029
HALLMARK_MTORC1_SIGNALING	−0.6054	−1.9889	0.0019	0.0072	0.029
HALLMARK_DNA_REPAIR	−0.5881	−1.9831	0.0019	0.007	0.031
HALLMARK_APOPTOSIS	−0.4891	−1.8768	0.0021	0.0227	0.096
HALLMARK_UNFOLDED_PROTEIN_RESPONSE	−0.5627	−1.8759	0.0019	0.0209	0.097
HALLMARK_MYC_TARGETS_V1	−0.683	−1.8714	0.0059	0.0204	0.104
HALLMARK_HEME_METABOLISM	−0.4404	−1.8452	0.002	0.0223	0.112
HALLMARK_UV_RESPONSE_DN	−0.5057	−1.8305	0.0101	0.0233	0.125
HALLMARK_ADIPOGENESIS	−0.4763	−1.8229	0.0059	0.0231	0.128
HALLMARK_TGF_BETA_SIGNALING	−0.5571	−1.8106	0.0165	0.0245	0.131
HALLMARK_IL2_STAT5_SIGNALING	−0.436	−1.8089	0.0041	0.024	0.137
HALLMARK_UV_RESPONSE_UP	−0.4501	−1.7753	0	0.0298	0.171
HALLMARK_OXIDATIVE_PHOSPHORYLATION	−0.576	−1.734	0.0429	0.0388	0.216
HALLMARK_HYPOXIA	−0.4384	−1.7291	0.0204	0.0383	0.223

## Discussion

As a fundamental biological process of energy metabolism and material recycling, the role, process, and function of autophagy in the heterogeneous metabolic pattern of tumors have long been widely concerned. Cellular autophagy itself can be hijacked by tumor cells for the synthesis of biomacromolecules required for tumor cell proliferation (16). The metabolic pattern that tumor cells distinguish from normal cells is termed aerobic glycolysis or is described as the Warburg effect. This metabolic pattern is characterized by enhanced uptake of glucose and amino acids by tumor cells, opportunistic nutrient utilization patterns, differential glycolysis/TCA cycle pathways, increased demand for nitrogen elements, a metabolite driven pattern of gene regulation, and interaction with metabolites in the tumor microenvironment, features that are widely used for tumor-specific targeted drug development (17). Autophagy extensively cross-talk these processes, serving as a ‘bridge’ for the activation and synthesis of most metabolic pathways and signaling molecules within the cell, playing diverse roles at different stages of tumor initiation and progression (18). Since tumorigenesis depends on the accumulation of damaged intracellular biomacromolecules, especially DNA, and autophagy facilitates the clearance of intracellular ROS and damaged organelles, in the early malignant biological events of tumorigenesis, autophagy is thought to be able to inhibit the tumorigenesis of cancer cells (19, 20). Whereas the maintenance role of autophagy for such a heterogeneous metabolic pattern is manifested when tumors progress. From one classic scenario, defects in basal autophagy in tumor cells limit nutrient supply for recycling of intracellular constituents. Although scholars have gained some understanding of the promoting role of autophagy on tumor survival, little is known about how nutrient deprivation caused by impaired autophagy affects metabolically driven tumor progression (21). The work of Lin et al. (22) showed that the survival cycle of KRAS-G12V driven tumor-bearing mice was extended by 38% when the autophagy-related gene ATG5 was conditionally knocked out (ATG5-ko), and ATG5 knockout tumor cells exhibited decreased mitochondrial function and increased mitochondrial fragmentation. Interestingly, ATG5 knockout tumor cells, in spite of the compensatory overexpression of asparagine synthetase (ASNs), displayed a lack of expression in the tumor cells of asparagine, a nonessential amino acid, in the metabolite profile. Moreover, inhibition and downregulation of autophagy and ASNs reduced KRAS-G12V driven tumor cell proliferation, migration, and invasion ability, and this ability could be rescued by asparagine supplementation or knockdown of the mitochondrial fission factor (MFF).

In addition, our single gene set enrichment analysis performed in the pan-cancer analysis of ATG5 using high and low ATG5 expression as a signature of sample subgroups showed that ATG5 can participate in a wide range of metabolic pathways and biosynthesis, including glycolysis, PI3K-Akt-mTOR signaling, mTORC1 signaling, p53 signaling, pyrimidine metabolism, TGF-β signaling, IL2-STAT5 signaling, and oxidative phosphorylation. And the highest enrichment scores of these signaling pathways all fell in the high expression area of ATG5. This suggests that high ATG5 expression is involved in the positive regulation of these signaling pathways, and ATG5 may play a pathological role in promoting aerobic glycolysis, maintaining tumor cell viability, and promoting tumor cell proliferation by driving the upregulation of these metabolic pathways and signal transduction pathways.

If the results presented above justify the idea that autophagy determines the metabolism of tumor heterogeneity, then the latest report by Yamamoto et al. (23), showed that autophagy can promote the immune escape of pancreatic cancer cells by degrading MHC-I, was sufficient to argue that the conjecture that autophagy is associated with tumor immune escape is well-founded. Notably, in addition to focusing on CD8^+^ T cells, this work observed changes in other immune cells such as MDSCs, CD4^+^ T cells, and CD103^+^ DCS upon autophagy inhibition, which also explains the results exhibited by our correlation analysis between ATG5 and tumor immune infiltration, suggesting that autophagy-mediated immune escape from tumors may be ubiquitously through signaling pathways in different tumor immune cells.

Naturally, blocking the autophagy pathway can be considered as a core strategy in tumor-targeted therapy. Indeed, a series of clinical studies based on the therapeutic application of the autophagy inhibitor chloroquine in tumors have been initiated and it was demonstrated that in some kinds of tumors, inhibition of autophagy contributes to the survival of tumor patients (24, 25). However, the complexity of autophagy itself determines, and its targets are characterized by diversity. How to select the optimal autophagy targeting gene will be a central question in tumor-targeted therapy that blocks autophagy.

ATG5, as an autophagy gene, played an important role in the process of autophagosome formation and autophagic flux and regulated multiple biological behaviors of tumors (3–8). The ATG5 protein was first discovered in the yeast system to be involved in the early stages of autophagosome formation, and the atg5-atg12 conjugation system plays an important role in the formation of the autophagosomal membrane and the recruitment of LC3. A series of studies have shown that ATG5 can determine autophagy progression and cell fate determination by affecting protein ubiquitination and autophagy-lysosome formation (26, 27). These regulatory roles of ATG5 were also validated again by our pan-cancer analysis, in addition to ATG5 mediated activation of oxidative phosphorylation, p53 signaling pathway, and others reported. But more biological processes such as IL-2/STAT5 and cross-talk with ATG5 are still open questions to be investigated. The significance of our work is that the crosstalk of these possible signaling pathways was prospectively revealed, providing bioinformatics and computational biology based insights for further understanding the role played by ATG5 in tumor metabolism and immune escape.

## Data Availability Statement

The original contributions presented in the study are included in the article/supplementary material. Further inquiries can be directed to the corresponding authors.

## Author Contributions

CXX and YSZ have contributed equally to this work. MX, YCZ, and YXZ conceptualized and designed the study. YXZ, WQC, and HZ performed the bioinformatics analysis. CXX and YXZ wrote the first draft of the manuscript. All authors wrote sections of the manuscript. All authors contributed to the article and approved the submitted version.

## Conflict of Interest

YXZ was employed by Biotrans Technology Co., LTD.

The remaining authors declare that the research was conducted in the absence of any commercial or financial relationships that could be construed as a potential conflict of interest.
